# Failed Trial Without Catheter Post Rezūm in Non‐Catheter Dependent Patients: Risk Factors From the Canadian Rezūm Registry

**DOI:** 10.1111/luts.70054

**Published:** 2026-03-06

**Authors:** Omar Buksh, Roseanne Ferreira, Mario Henrique Bitar Siqueira, Naeem Bhojani, Bilal Chughtai, Kevin C. Zorn, Dean S. Elterman

**Affiliations:** ^1^ Division of Urology, Department of Surgery University of Toronto Toronto Ontario Canada; ^2^ Division of Urology Centre Hospitalier de l’Université de Montréal (CHUM) Montréal Quebec Canada; ^3^ Plainview Hospital, Smith Institute of Urology, Northwell Health New York New York USA; ^4^ BPH Canada, Prostate Surgical Institute Montreal Quebec Canada

**Keywords:** benign prostatic hyperplasia, minimally invasive surgical therapy, Rezum, urinary retention

## Abstract

**Introduction:**

Rezūm has become an increasingly popular surgical approach for treating bladder outlet obstruction. Despite the practice of routinely placing a Foley catheter, successful TWOC (Trial Without Catheter) remains variable. This study seeks to identify the TWOC failure incidence and risk factors to inform clinical decisions and enhance patient counseling.

**Methods:**

We conducted a retrospective review of non‐catheter dependent patients who underwent Rezūm therapy between April 2019 and June 2023 in two high‐volume Canadian centers. All patients received a urinary catheter post‐treatment. International Prostate Symptom Score (IPSS), QoL, Qmax, and PVR were evaluated. Risk factors for TWOC failure were determined through logistic regression.

**Results:**

Out of 406 patients, 99 patients (24.4%) failed TWOC on the first attempt. Median time to TWOC was 7 days (range 3–30 days). Successful and failure groups had no significant difference in terms of average catheterization time, baseline prostate volume, PVR, or total number of injections per prostate volume ratio. A higher rate of UTIs was observed in the TWOC failure group (10.6% vs. 2.6%, *p* = 0.003). During the 12‐month follow‐up period, 12 patients developed episodes of urinary retention following Rezūm therapy. Baseline PVR was not a risk factor for failed TWOC in our cohort (*p* = 0.95). Higher IPSS was a predictor for TWOC failure (OR 1.04 95% CI: 1.01–1.08).

**Conclusions:**

About one in four patients failed the first TWOC after Rezūm therapy. High baseline IPSS was identified as the only risk factor for TWOC failure in our cohort. Despite this, overall urinary symptoms improved in all patients.

## 1 Introduction

Benign Prostatic Hyperplasia (BPH) is a prevalent condition affecting aging men, particularly beyond the age of 50, with up to 80% experiencing Lower Urinary Tract Symptoms (LUTS) due to BPH by their eighth decade [1]. In Canada, the prevalence of BPH in men aged 50 and older exceeds 1 million [2]. LUTS encompass various urinary issues, such as urgency, frequency, and difficulty in initiating urination or a weak stream [3]. Initially managed through lifestyle changes and medication, symptomatic BPH often necessitates surgical intervention, with Transurethral Resection of the Prostate (TURP) traditionally being the gold standard [4].

TURP's efficacy is well‐established, but its acute and long‐term effects have been extensively documented. Its dominance in BPH surgical treatment, which was over 80% up to 1999, declined to 39% by 2005, largely due to advancements in newer, more appealing BPH procedures [5]. Among these, Minimally Invasive Surgical Techniques (MIST) have emerged as alternatives to TURP. The Rezum Convective Water Vapor Thermal Therapy (CWVTT‐Rezum) (Rezum System, Boston Scientific, Marlborough, Massachusetts), which uses radiofrequency water vapor thermal energy to induce cell necrosis in the prostatic lobes, is one such MIST gaining popularity [6]. This technique has proven effective and safe for treating LUTS/BPH, is particularly appealing due to its suitability for a range of patients, including those with urinary retention, catheter dependency, varying prostate sizes, and the presence of median lobes [7].

Rezum therapy's increasing preference for LUTS/BPH treatment is partly due to its feasibility in an office setting, with patients generally leaving shortly post‐procedure with a Foley catheter [8]. The removal of this catheter is at the urologist's discretion, with no consensus on the optimal timing, typically ranging from 3–8 days post‐operation [8, 9]. However, a subset of patients experience TWOC (Trial Without Catheter) failure post‐Rezum therapy, raising concerns for both patients and urologists about the risk factors for failed void trials and their impact on retreatment rates. This variability in TWOC failure rates [6] and unclear risk factors underscores the necessity for a comprehensive understanding of these factors. Therefore, our study aims to identify TWOC failure risk factors in a large, multicentric, real‐world cohort, providing critical insights for patient counseling, and evidence‐based decisions regarding postoperative catheter duration.

## 2 Methods

### 2.1 Study Subjects

A convective water vapor thermal therapy (CWVTT‐Rezum) (Rezum System, Boston Scientific, Marlborough, Massachusetts) prospective registry was established at two high‐volume Canadian centers. Institutional ethics board approval was obtained at each center. We retrospectively evaluated charts from patients who underwent Rezum therapy between April 2019 and June 2023. Patients who were catheter‐dependent at the time of Rezum therapy or had confirmed neurogenic bladder were excluded from the study.

### 2.2 Treatment Procedure

The procedure of Rezum water vapor therapy was conducted following the methods outlined in previous studies [9]. The system utilizes thermal energy from water vapor conveyed through a retractable needle accompanied by a saline flush. Each application of 9 s of water vapor ablates contiguous regions of the prostatic tissue following the urethra's natural gradient, considering each patient's gland's shape and position, including the median lobe. The number of injections was decided by the surgeon's discretion after analyzing the patient's prostatic anatomy and patients' interest in preserving ejaculatory function. Rezum was carried out in an office setting as outpatient services under sedation, Penthrox inhalation, or spinal block. Patients were discharged the same day with a Foley in situ. As per protocol, duration of catheter was left at the surgeon's discretion and was generally standardized to 7 days.

### 2.3 Data Collection and Outcome Measures

Patients were followed at 1‐, 3‐, 6‐, 12 months and yearly after that. Patients' characteristics, prostatic volume, International Prostate Symptom Score (IPSS) and IPSS quality of life (QoL) subscale, International Index of Erectile Function (IIEF‐15), maximum urinary flow rate (Qmax), post‐void residual (PVR), PSA, number of Rezum injections, complications, and BPH medication usage were evaluated at baseline and follow‐up. Failed TWOC was defined as the inability to spontaneously void after catheter removal. Postoperative urinary tract infection (UTI) was defined as the presence of urinary symptoms and a positive urine culture occurring during the postoperative catheterization period, prior to catheter removal and the first TWOC attempt.

### 2.4 Statistical Analysis

Patients' characteristics and assessments were reported descriptively. The study conducted a thorough comparison of demographic and clinical features between patients with successful and failed TWOC. Continuous variables were analyzed using either the Student's *t*‐test or the Mann–Whitney U test, ANOVA or Kruskal–Wallis based on their distribution. Categorical variables were compared using Fisher's exact test. A logistic regression model was applied for both univariable and multivariable analyses to identify risk factors for TWOC failure. Variables showing significant associations in the univariate analysis were included in the multivariate model and adjusted for confounding factors like age, comorbidities, and baseline prostate size. A subgroup analysis was planned for patients with successful versus failed TWOC. All statistical analyses were performed using Stata 18BE, and a two‐sided *p*‐value of less than 0.05 was considered statistically significant.

## 3 Results

### 3.1 Overall

Out of 480 patients treated with Rezum during this period, 406 patients met eligibility criteria. Patients underwent surgery at a mean age of 68.0 ± 8.4 years and had a median prostatic volume of 64 (IQR 47–93) cc. A total of 143 (35.3%) patients had prostate size greater than 80 mL; 262 (64.5%) patients had a median lobe. The median cohort PVR was 107 (IQR 31–216) ml. In terms of comorbidities, 48 (11.8%) had a history of previous urinary retention, 29 (7.1%) had diabetes, 117 (28.8%) had hypertension, and 14 (3.4%) had depression Table 1.

**TABLE 1:** Characteristics between successful and failed TWOC patients.

	Total	Successful TWOC	Failed TWOC	*p*
	*N* = 406	*N* = 307	*N* = 99	
Age	68.3 (62.2–73.6)	68.2 (62.4–74)	68.7 (60.7–72.2)	0.34
Median lobe, *n* (%)	262 (64.5%)	203 (66.1%)	59 (59.6%)	0.28
Diabetes	29 (7.1%)	22 (7.2%)	7 (7.1%)	0.97
Hypertension	117 (28.8%)	88 (28.7%)	29 (29.3%)	0.9
History of previous urinary retention	48 (11.8%)	36 (11.7%)	12 (12.1%)	1
BPH medication usage, *n* (%)				
Alpha‐blockers	55 (13.5%)	38 (12.4%)	17 (17.2%)	0.16
5‐ARIs	69 (17.0%)	51 (16.6%)	18 (18.2%)	
Alpha‐blockers + 5‐ARIs	24 (5.9%)	16 (5.2%)	8 (8.1%)	
PDE5 only	18 (4.4%)	17 (5.5%)	1 (1.0%)	
Prostate volume, mL	64 (47–93)	65 (47–93)	60.5 (46.5–92.5)	0.43
Prostate volume, *n* (%)				
< 80 cc	26 (64.7%)	194 (63.2%)	68 (69.4%)	0.28
> 80 cc	143 (35.3%)	113 (36.8%)	30 (30.6%)	
PSA, ng/mL	3 (1.6–5.2)	3 (1.7–5.4)	2.99 (1.4–4.7)	0.46
Qmax, mL/s	7.6 (5–11.65)	7.9 (5–12)	7.2 (4–10)	0.2
PVR, mL	107 (31–216)	100 (28–206)	123.5 (47–237)	0.35
IPSS	23 (18–26.5)	22 (18–26)	24 (19–28)	0.047
QoL	5 (4–5)	5 (4–5)	5 (4–6)	0.17

All patients were discharged home with a catheter in situ. Overall, 99 patients (24.4%) failed TWOC on the first attempt post‐procedure. The median time to TWOC was 7 (IQR 7–13) days. There was no difference between the length of catheter stay for patients with successful or failed TWOC (9.4 ± 5.1 vs. 9.5 ± 4.6 days, *p* = 0.79). Patients with a history of urinary retention were catheterized for nearly twice as long as those without prior retention (14.1 ± 7.4 vs. 8.8 ± 4.2 days, *p* < 0.001). Length of catheter stay also varied based on prostatic volume: glands < 80 cc were catheterized for 7.7 ± 3.5 days, 80–149 cc glands for 11.5 ± 5.0 days and glands > 150 cc for 19.1 ± 8.0 days (corrected *p* < 0.001 for all comparisons).

Patients received a median number of 10 [7, 8, 9, 10, 11, 12, 13] injections, with 1.4 (IQR 1.1–1.7) injections per 10 mL of prostatic volume. There was no difference in the number of injections/10 mL prostatic volume between patients with a history of retention or not (*p* = 0.33). The median number of injections administered to the median lobe was 0 (range 0–3). There was no statistically significant difference between the two groups (*p* = 0.41). There were no Clavien–Dindo grade ≥ 3 postoperative complications. 18 (4.5%) patients developed UTIs Table 2.

**TABLE 2:** Rezum characteristics between successful and failed TWOC patients.

	Total	Successful TWOC	Failed TWOC	*p*
	*N* = 406	*N* = 307	*N* = 99	
Total number of injections—median (min–max)	10 (7–13)	10 (7–13)	9 (7–13)	0.24
Median lobe injection—median (min–max)	0 (0–3)	0 (0–3)	0 (0–2)	0.41
Injection/prostate volume ratio (per 10 cc)	1.4 (1.1–1.7)	1.4 (1.1–1.7)	1.4 (1.1–1.8)	0.89
Patients experiencing urinary retention, *n* (%)	12 (2.9%)	4 (1.3%)	8 (8.1%)	0.002
Time to retention, months	9.5 ± 10.8	21 ± 1.5	3.7 ± 3.7	0.002

### 3.2 Successful vs. Failed TWOC

Successful and failure groups had no significant differences in terms of average baseline prostate volume (*p* = 0.288), PVR (*p* = 0.35) or total number of injections per prostate volume ratio (*p* = 0.89). There was no significant difference between failed TWOC in patients with different baseline prostatic volumes [68 (25.9%) glands < 80 cc, 28 (21.9%) glands 80–149 cc and 2 (13.3%) glands > 150 cc, *p* = 0.479]. Demographics and procedure characteristics comparison between successful and failed groups can be found in Tables 1 and 2. Regarding postoperative complications, a higher rate of UTIs was observed in the TWOC failure group (10.6% vs. 2.6%, *p* = 0.003). The presence of UTI was found to significantly increase the predictive failure of TWOC (OR 1.64, 95% CI 1.59–1.69). Of the 99 patients who failed the initial TWOC, 9 (9.1%) subsequently underwent GreenLight laser therapy, 2 (2.0%) underwent TURP, 1 (1.0%) received iTIND, and 1 (1.0%) required CIC. The remaining 86 patients (86.9%) successfully passed the second TWOC.

During follow‐up, 12 (3%) patients experienced urinary retention. Patients who failed first TWOC were 7 times more likely to present with retention during follow‐up (OR 7.0 95% CI 2.1–23.9, *p* = 0.002). Patients failing their TWOC experienced retention sooner at a mean time to retention of 3.7 ± 3.7 months vs. 21 ± 1.5 months in the successful TWOC group (*p* = 0.002). Four patients required Clean Intermittent Catheterization (CIC) for adequate voiding (two in each TWOC group). There was no association between presenting an episode of urinary retention and requiring CIC (*p* = 0.11). Furthermore, 19 (4.7%) patients required re‐treatment. There was no difference between treatment failure rates between successful vs. failed TWOC groups (4.2% vs. 6.4%, *p* = 0.409, respectively) nor the difference between time to re‐treatment between groups (successful: 18.5 ± 10.2 vs. failed: 21 ± 13.0 months, *p* = 0.650) Table 2.

### 3.3 Risk Factors for TWOC

**FIGURE 1:**
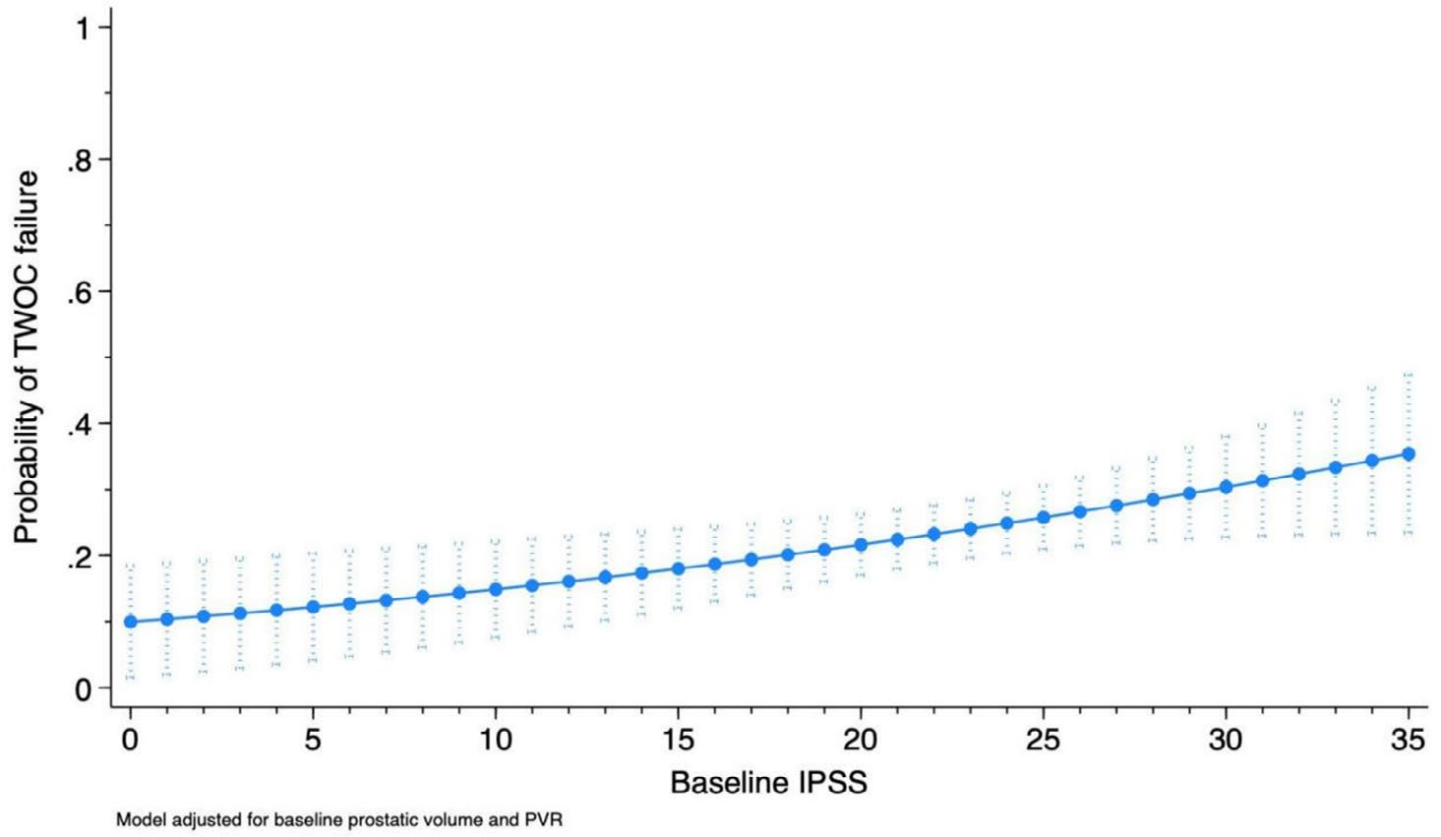
Probability of TWOC failure as a function of baseline IPSS adjusted for prostatic volume and baseline PVR. Predicted probability of TWOC failure according to baseline IPSS, adjusted for prostate volume and baseline PVR. The line represents model‐derived estimates, and vertical bars indicate 95% confidence intervals.

On univariable analysis, multiple potential risk factors were analyzed. Higher IPSS and UTI were predictors for TWOC failure in our cohort (OR 1.04 95% CI: 1.01–1.08), (OR 1.64, 95% CI 1.59–1.69), respectively. However, baseline PVR was not a risk factor for failed TWOC in our cohort (*p* = 0.95). Table 3 highlights the odds of TWOC failure based on potential risk factors. On multivariable analysis, IPSS and UTI remained the only independent predictors of TWOC failure. Figure 1 highlights the probability of TWOC failure for different IPSS values adjusted for prostatic volume and PVR. Particularly, patients with IPSS scores greater than 23 points have 71% higher odds of experiencing TOV failure compared to those with a lower IPSS score (OR 1.71, 95% CI 1.01–2.81). When stratified by baseline IPSS, patients with IPSS < 23 had lower rates of TWOC failure compared to higher scores (19.1% vs. 27.9%, *p* = 0.043), as well as lower baseline PVR (129.3 ± 10.1 mL vs. 166.2 ± 12.8 mL, *p* = 0.027). There was no difference in length of catheter duration (*p* = 0.396) or baseline prostatic volume (*p* = 0.089) between IPSS groups.

**TABLE 3:** Risk factors of TWOC.

Risk factor	Univariable analysis	*p*	Multivariable analysis	*p*
	OR (95% CI)		OR (95% CI)	
Age, years, median	0.98 (0.96–1.00)	0.213		
History of urinary retention	1.04 (0.52–2.08)	0.916		
Prostate Volume	1.00 (0.99–1.00)	0.288	1.00 (0.99–1.00)	0.584
Median lobe	0.80 (0.50–1.27)	0.348		
PSA, ng/mL	1.02 (0.97–1.08)	0.355		
PVR, mL	1.00 (0.99–1.00)	0.954	1.00 (1.00–1.00)	0.706
Qmax, mL/s	0.97 (0.92–1.01)	0.181		
IPSS	1.04 (1.00–1.08)	0.042	1.05 (1.01–1.09)	0.023
IIEF	0.99 (0.98–1.01)	0.490		
Total number injections	0.96 (0.91–1.01)	0.142		
Injections/10 cc prostate	0.99 (0.65–1.51)	0.956		
Length of catheter duration, days	1.00 (0.96–1.05)	0.795		

## 4 Discussion

This study evaluated the rate and risk factors for TWOC failure in patients who were catheter‐independent at the time of Rezum that were followed up to 12 months. We observed a TWOC failure of 24.4%, with baseline IPSS and the presence of a UTI being strong independent risk factors for failure, with patients with IPSS > 23 having 70% higher odds of experiencing TWOC failure than those with lower IPSS scores. Length of catheter stay or baseline PVR was not significantly different between successful and failed groups. Patients with TWOC failure were more likely to develop UTIs but ultimately had no difference in catheter independence and treatment failure rates than patients with successful TWOC.

Following Rezum therapy, edema and inflammation are anticipated, necessitating the pre‐emptive placement of a Foley catheter to mitigate the risk of urinary retention. Currently, there are no formal guidelines recommended by urological societies regarding the duration of catheterization post gland ablation, nor are there established risk factors to inform this decision. In our study, catheterization duration was determined by surgeon discretion, influenced primarily by prostatic size and patients' history of urinary retention. Specifically, patients with no previous retention history and prostatic glands smaller than 80 cc typically had a median catheterization duration of about 7 days, while those with a history of retention experienced approximately 14 days of catheterization. Patients with larger glands also underwent longer periods of catheterization. Overall, the median time to the first post‐procedure trial without catheter (TWOC) was 7 (IQR 7–13) days. Notably, there was no significant difference in catheter duration between groups with failed or successful TWOC. Prior studies on Rezum's functional outcomes report a range of catheterization durations from 3.0 to 32.2 days, with an average of around 3.4 days for patients without a history of retention [7, 9, 10]. A cohort study of patients with an average prostatic volume of 48 cc, showing a mean catheterization time of 4 ± 1.7 days, found no significant difference in catheter duration between successful and failed TWOC (4.1 ± 1.7 vs. 3.4 ± 1.3 days, *p* = 0.11) [11]. Length of catheter duration was not a risk factor for catheter failure in either of cohorts [11, 12]. This suggests that longer catheterization does not necessarily correlate with improved outcomes, even for durations as short as 4 days.

In our cohort, the TWOC failure rate was 24.4%, is similar to previously reported rates. The independent risk factor for failed TWOC in our study were high IPSS and UTI. A preceding study noted a 12.1% failure rate in TWOC post‐Rezum, where patients were catheterized for a median of 5 days postoperatively [12]. This previous cohort, composed of patients with lower median baseline IPSS scores (18, IQR 11–24), contrasts with our cohort's higher IPSS values (23, IQR 18–26.5), particularly among patients with failed TWOC (24, IQR 19–28) [12]. The correlation between higher IPSS and TWOC failure was consistent in both studies, offering an explanation for the higher failure rates observed in our cohort. Felice et al.'s study, which reported a TWOC failure rate of 17%, also supports this finding [11]. Their patient group, with failed TWOC, had higher IPSS scores (24, IQR 18–27), more akin to those in our study, potentially accounting for their elevated failure rates.

Additionally, PVR has been identified as an independent risk factor for TWOC failure in both Rezum therapy and other BPH MIST modalities. In the studies by Babar and Felice, the failed groups exhibited significantly higher PVR volumes [11, 12]. Despite these findings, our data showed no significant difference in baseline PVR values between the successful and failed TWOC groups (123.5 mL [IQR 47–237] vs. 100 mL [IQR 28–206], *p* = 0.95). Felice et al. noted that the risk of failed TWOC increased by 2% for every 5‐mL increase in preoperative PVR [11]. However, baseline PVR was not identified as a predictive factor for TWOC failure in our cohort. One possible explanation relates to the distribution of PVR values within our population, where residual volumes were relatively elevated across both groups. This limited variability may have reduced its discriminatory capacity, suggesting that PVR may be a weaker predictor in cohorts with uniformly higher baseline residual volumes.

Given the likelihood that a higher PVR indicates preoperative bladder decompensation [13], PVR has a very weak correlation to IPSS [14] and the absence of an interaction between these two variables, it's plausible that higher IPSS values might be a surrogate of underlying bladder dysfunction or presence of compensatory mechanisms, rather than TWOC being directly influenced by PVR. Patients who failed TWOC exhibited a higher incidence of detrusor underactivity and reduced bladder voiding efficiency [11]. However, due to the lack of comprehensive urodynamic data for our patients, it remains unclear how different signs of bladder dysfunction are appear as significant TWOC failure risk factors for patients with a range of dysfunction severity. We cannot exclude the possibility that PVR might be the first sign of bladder decompensation and thus only significant TWOC risk factor to mild patients.

Despite variations in catheter duration post‐Rezum based on prostatic size, our study found no difference in TWOC failure rates relative to baseline prostatic volumes. This finding aligns with Babar et al.'s study, which reported comparable TWOC failure rates regardless of prostatic volume size [12]. Additionally, our data indicated a higher incidence of UTIs in patients who failed their TWOC. Multivariate analyses revealed that catheterization extending beyond 2 days postoperatively is associated with a higher likelihood of developing an in‐hospital UTI (HR 1.21, 95% CI 1.04–1.41) [15]. UTIs were also linked to increased rates of acute urinary retention [16].

While alternative treatments like Urolift exist, which do not show an increase in urinary retention rates, comparative studies have consistently demonstrated Rezum's superior outcomes in terms of improved Qmax and notably lower long‐term re‐treatment rates [17]. Despite a 24.4% TWOC failure rate in our cohort, failed TWOC was not associated with higher rates of treatment failure. Therefore, patients should be informed about the potential for TWOC failure, taking into consideration their individual risk factor profiles and the lack of association with overall treatment success.

This study, however, is not without limitations. Firstly, the duration of catheterization was determined by the surgeon based on the patient's gland anatomy, without a standardized protocol. Secondly, the small rate of TWOC failure in our study may have been underpowered to identify significant differences between other potential risk factors. Thirdly, a detailed breakdown of the IPSS score was not available, which would have aided in identifying specific phenotypes driving these scores or the role of storage/voiding score ratios in TWOC failure rates. Lastly, although patients with neurogenic bladder were excluded, we lacked comprehensive data on bladder health, which could influence voiding function. Despite these limitations, our study represents the largest real‐world cohort of Rezum patients to date, assessing risk factors for catheter failure across a diverse range of severities, prostate sizes, and LUTS severities as encountered in a urology clinic. The data, collected from two centers, enhances the generalizability of our findings and offers valuable insights for the future development of a post‐Rezum catheterization protocol. Our findings suggest that pre‐procedure IPSS could guide catheter duration decisions, with patients presenting an IPSS greater than 23 potentially benefiting from a longer catheterization period.

Prospective studies with standardized catheter management protocols are warranted to reduce practice variability and more accurately define optimal TWOC timing. Moreover, incorporation of comprehensive urodynamic parameters may better delineate the underlying mechanisms driving early postoperative voiding failure and refine predictive models beyond symptom scores and residual volumes alone.

## 5 Conclusion

Approximately 25% of our patients did not successfully pass the initial trial without a catheter following Rezūm therapy. Notably, a high baseline IPSS and UTI emerged as identifiable risk factors for TWOC failure in our patient group, with patients with IPSS > 23 presenting 70% higher odds of TWOC failure. However, regardless of these initial challenges, there was a universal improvement in urinary symptoms among all patients' post‐treatment with a low retreatment rate of 4.7% regardless of initial TWOC status. This finding underscores the overall effectiveness of Rezūm therapy in managing urinary symptoms, even in cases where initial TWOC attempts are unsuccessful.

## Funding

Funding for the Rezūm registry was provided by Boston Scientific.

## Conflicts of Interest

Dr. Elterman is a consultant/investigator for Boston Scientific, Procept Biorobotics, Olympus, Urotronic, Prodeon, and Zenflow. Dr. Chughtai is a consultant for Boston Scientific, Olympus, Procept, Prodeon. Dr. Zorn is a consultant/investigator for Boston Scientific and Procept BioRobotics. Dr. Bhojani is a consultant/investigator for Boston Scientific, Procept BioRobotics, and Olympus. The remaining authors declare no conflicts of interest.

## Data Availability

The data that support the findings of this study are available on request from the corresponding author. The data are not publicly available due to privacy or ethical restrictions.
